# Highly potent silver-organoalkoxysilane antimicrobial porous nanomembrane

**DOI:** 10.1186/1556-276X-8-164

**Published:** 2013-04-10

**Authors:** Sirajo Umar, Yuanfeng Liu, Yiguang Wu, Guangtao Li, Jiabo Ding, Runsong Xiong, Jinchun Chen

**Affiliations:** 1 College of Life Science and Technology, Beijing University of Chemical Technology, Beijing 100029, China; 2 Department of Chemistry, Tsinghua University, Beijing 100084, China; 3 China Institute of Veterinary Drug Control, Beijing 100081, China

**Keywords:** Polyvinylpyrrolidone (PVP), Silver, Nanoparticles, Antimicrobial, Composite fibers

## Abstract

We used a simple electrospinning technique to fabricate a highly potent silver-organoalkoxysilane antimicrobial composite from AgNO_3_-polyvinylpyrrolidone (PVP)/3-aminopropyltrimethoxysilane (APTMS)/tetraethoxysilane (TEOS) solution. Spectroscopic and microscopic analyses of the composite showed that the fibers contain an organoalkoxysilane ‘skeleton,’ 0.18 molecules/nm^2^ surface amino groups, and highly dispersed and uniformly distributed silver nanoparticles (5 nm in size). Incorporation of organoalkoxysilanes is highly beneficial to the antimicrobial mat as (1) amino groups of APTMS are adhesive and biocidal to microorganisms, (2) polycondensation of APTMS and TEOS increases the membrane’s surface area by forming silicon bonds that stabilize fibers and form a composite mat with membranous structure and high porosity, and (3) the organoalkoxysilanes are also instrumental to the synthesis of the very small-sized and highly dispersed silver metal particles in the fiber mat. Antimicrobial property of the composite was evaluated by disk diffusion, minimum inhibition concentration (MIC), kinetic, and extended use assays on bacteria (*Escherichia coli*, *Bacillus anthracis*, *Staphylococcus aureus*, and *Brucella suis*), a fungus (*Aspergillus niger*), and the Newcastle disease virus. The membrane shows quick and sustained broad-spectrum antimicrobial activity. Only 0.3 mg of fibers is required to achieve MIC against all the test organisms. Bacteria are inhibited within 30 min of contact, and the fibers can be used repeatedly. The composite is silver efficient and environment friendly, and its membranous structure is suitable for many practical applications as in air filters, antimicrobial linen, coatings, bioadhesives, and biofilms.

## Background

For the purpose of achieving higher antimicrobial functionality and circumventing the solubility problems associated with silver-based antimicrobial materials, duo- and multiaction-component composites harboring silver and its halides with other antimicrobial agents were fabricated [1-3]. Although these new materials showed a step forward in terms of antimicrobial effectiveness, some of them are of limited practical applications as they exist in powder form, and in some instances, fabrication procedures were cumbersome involving many steps [4]. Hence, the need for fabrication of highly effective antimicrobial composites suitable for many practical applications is still a challenge. Both the ionic and metallic forms of silver are believed to have antimicrobial potency, though potency of metallic particles is considered superior. Antimicrobial activity of composites’ silver particles has a close relationship with the size and degree of dispersion of the particles. Generally, bigger silver particles are less effective compared to smaller particles, and aggregation of silver particles causes deterioration of antimicrobial activity in composite materials [5,6]. High surface area-to-volume ratio of antimicrobial composites is also beneficial to the efficiency of incorporated antimicrobial agents and improves the overall antimicrobial activity of the composites [7]. In this research, we used a simple electrospinning technique to prepare a highly potent silver-organoalkoxysilane antimicrobial fiber mat from AgNO_3_-polyvinylpyrrolidone (PVP)/3-aminopropyltrimethoxysilane (APTMS)/tetraethoxysilane (TEOS) solution. The technique allows fine-tuning and fabrication of fibrous composite from a viscous solution. Organoalkoxysilanes (APTMS and TEOS) were chosen because they are both liquids and easy to use in electrospinning. With electrospinning and organoalkoxysilanes, we were able to condition the fibrous composite with high surface area-to-volume ratio and monodispersed, small-sized silver particles. Moreover, in addition to silver particles, amino groups of APTMS also act as antimicrobial agent. Majority of reports on antimicrobial silver-nanofiber composites were conducted on two common bacterial strains: *Escherichia coli* and *Staphylococcus aureus*, and highly pathogenic bacteria like anthrax and brucella, and other microbial groups such as fungi and viruses are often avoided. We tend to have a comprehensive antimicrobial evaluation of the composite in this report. Hence, in addition to the common bacteria, pathogenic species (anthrax and brucella), a fungus (*Aspergillus niger*), and a virus (Newcastle disease virus) were also included as test organisms in this work.

## Methods

### Materials

PVP (K1300) was obtained from Aldrich (St. Louis, MO, USA), APTMS (97%) from Alfa Aesar (Ward Hill, MA, USA), and TEOS (98%) from J&K Chemica (Beijing, China). Ethanol (99.7%) and silver nitrate were available from Beijing Chemical Works (Beijing, China). Microbial culture media were purchased from Becton Dickenson (Franklin Lakes, NJ, USA). All reagents were used as received. *E. coli* JM109 was obtained from our laboratory, and *S. aureus* ATCC6358P and the fungus *A. niger* ATCC10864 were kind donations from Professors Zhang Peng and Zhang Shurong of the College of Life Science and Technology, Beijing University of Chemical Technology. *Brucella suis* strain 2 CVCC70-502 (China Veterinary Culture Collection 502), *Bacillus anthracis* (CVCC40-221), and Newcastle disease virus LaSota strain were obtained from China Institute of Veterinary Drug Control.

### Nanomembrane fabrication

A 5 wt.% (relative to the weight of PVP) AgNO_3_ was dissolved in 11.3 g of ethanol followed by addition and dissolution of 2 g of PVP so that its concentration is kept at 15 wt.%. Electrospinning of the feed solution was made by adding in the following sequence AgNO_3_-PVP solution/APTMS/TEOS (*v*/*v*) 3:2:1 and was kept stirring for 30 min at room temperature. The feed solution was loaded into a plastic syringe equipped with a flat-tip stainless steel needle connected to a positive electrode with high voltage supply capable of generating up to 40 kV. The counter electrode was a flat aluminum foil placed 10 cm away from the needle’s tip. With a working voltage of 13 kV, jets of nanofibers were deposited on the foil, forming a white, shiny porous membrane. The as-spun fibers were left in air overnight for further hydrolysis of alkoxysilanes. Subsequently, the composite was aged by heating at 60°C and 90°C for 5 h each and at 120°C for 2 h. Positive experimental controls were fabricated from PVP/APTMS/TEOS (*v*/*v*) 3:2:1 without silver content and AgNO_3_-PVP/TEOS (*v*/*v*) 3:2 (with 5 wt.% AgNO_3_ relative to PVP) as described above.

### Instrumentations

Scanning electron microscopy (SEM) images of the fiber mat were recorded with Hitachi S-4700 (Chiyoda-ku, Japan). SEM acceleration voltage was 20 kV. The size and morphology of the products were studied by transmission electron microscopy (TEM; H-800) and high-resolution transmission electron microscopy (HR-TEM). Acceleration voltage for TEM was 200 kV. Fiber samples for TEM were made into powder and dispersed in acetone by ultrasonication, loaded on a carbon-coated copper grid, and then allowed to dry at room temperature before recording the micrographs. HR-TEM images were taken with JEOL JEM 2100 (Akishima-shi, Japan). Chemical identity of silver species in the composite was analyzed by X-ray photoelectron spectroscopy (XPS; Escalab 250, Thermo Fisher, Waltham, MA, USA). The X-ray excitation energy was 1,486 eV (Al Kα), and the spectra were recorded with a pass energy of 30 eV. The presence of organoalkoxysilanes was confirmed by nuclear magnetic resonance (NMR; Bruker TOPSPIN2.1, Madison, WI, USA). Density of the composite’s surface amino groups was obtained with a UV–vis spectrophotometer (UV-2450, Shimadzu, Kyoto, Japan), and silver ion release pattern was analyzed by inductively coupled plasma atomic emission spectroscopy (ICP/AES) with a sequential plasma spectrometer (ICPS-7500, Shimadzu).

### Determination of surface amino group density

The total amount of accessible primary amino groups of the AgNO_3_-PVP/APTMS/TEOS composite was determined with a modified surface imine formation procedure of Zhao et al. [8]. Pieces of the composite mat were carefully vacuum-dried at 343 K, sealed, and subjected to argon atmosphere. Then 30 mL of anhydrous ethanol containing 4-nitrobenzaldehyde (1 mg/mL) and catalytic amounts of acetic acid (0.75 μL) was added. The reaction was performed at 50°C for 3 h. The fibers were then washed with ethanol and sonicated in ethanol for 1 min before being vacuum-dried. Subsequently, the new imine-functionalized material was hydrolyzed in water (10 mL) containing acetic acid (0.25 μL) at 30°C for 1 h, and the regenerated imine concentration was determined by spectrophotometry at *λ* = 268.5 nm.

### Silver ion release rate

The concentration of silver ions released in phosphate-buffered saline (PBS; 5.4 g of sodium dihydrogen phosphate monohydrate and 8.66 g of anhydrous disodium hydrogen phosphate in 1 L of distilled water, pH 7.0) from AgNO_3_-PVP/APTMS/TEOS fibers was measured by ICP/AES [9]. One gram of composite sample was incubated in 100 mL of PBS at room temperature, and 5-mL solutions recovered at various times up to 80 h were analyzed by ICP/AES. A commercial ICP standard solution containing 50 ± 0.1 ppm of Ag^+^ was used for calibration.

### Antimicrobial tests

#### Culture conditions

All the organisms but brucella were cultivated in sterilized Luria Bertani (LB) broths (1% tryptone, 0.5% yeast extract, and 1% NaCl) on a 200-rpm shaking incubator at 37°C. Plating was done on LB 1% agar plates. Brucella strain was cultivated under the conditions mentioned above in tryptic soy broth (peptone from casein 17.0 g/L, peptone from soymeal 3.0 g/L, d-(+)-glucose 2.5 g/L, sodium chloride 5.0 g/L, and dipotassium hydrogen phosphate 2.5 g/L), and plating was done on tryptic soy agar (peptone from casein 15.0 g/L, peptone from soymeal 5.0 g/L, sodium chloride 5.0 g/L, and agar-agar 15.0 g/L). Plates are poured and allowed to cool for 24 h to ensure that they are completely dry before use. *A. niger* was cultured at 30°C.

#### Evaluation of antimicrobial activity

Prior to the experiment, all materials were accordingly sterilized by autoclaving, UV irradiation, or filtration. Antimicrobial activity of the AgNO_3_-PVP/APTMS/TEOS mat was evaluated on gram-negative bacteria (*B. anthracis* and *E. coli*), gram-positive bacteria (*S. aureus* and *B. suis*), and a fungus (*A. niger*) by qualitative and quantitative methods.

Qualitative evaluation was performed with a modified Kirby-Bauer assay [10]. A 6-mm-diameter rounded piece of the mat was placed on a LB agar growth plate seeded with 100 to 200 μL (approximate concentration of 5 × 10^7^ cfu/mL) overnight cultures of microorganisms then incubated for 12 h. Antibacterial activity was identified and estimated by a clear zone of inhibition in the indicator lawn around the fiber samples. PVP/APTMS/TEOS and AgNO_3_-PVP/TEOS served as positive controls and a clean filter paper as negative control. Sizes of inhibition zones were measured and digital images of the plates captured.

For minimum inhibition concentration (MIC) test, the inoculum was prepared by growing each microorganism in a liquid medium until a level of approximately 5 × 10^7^ cfu/mL was reached. Eight sterilized culture tubes were prepared, each containing 10 mL of fresh LB medium and 10 μL of microbial culture, and then 0, 0.3, 0.6, 0.9, 1.5, 3, and 6 mg of fiber mat pieces were, respectively, added into the tubes. The tubes were incubated for 24 h on a shaker, and then survival of bacteria was noted by visual inspection. To confirm whether the effect of the membrane was bacteriostatic or bacteriocidal, 100 μL was drawn from cultures that appeared to have little or no cell growth and then plated and incubated for 12 to 48 h for colony-forming unit (cfu) count. The composite MIC was calculated as the lowest concentration at which bacterial growth was inhibited. Quantity of active silver utilized by the MIC composite was analyzed by ICP/AES. All assays were carried out in triplicate.

Antimicrobial efficiency (kinetics) of AgNO_3_-PVP/APTMS/TEOS fibers was evaluated on *E. coli* by viable cell count method. Positive (PVP/APTMS/TEOS and AgNO_3_-PVP/TEOS) and negative (fiber-free *E. coli* suspension) controls were set up in this test. Composite pieces were incubated in a test tube containing 3 mL of 5 × 10^7^ cfu/mL *E. coli* suspension with continuous shaking. A 50-μL solution was removed from the tube as a function of contact time (min) and plated on LB agar plates to determine the number of surviving colonies. The plates were cultivated for 24 h, and numbers of viable colonies were counted to establish a function of percentage reduction versus contact time in minutes. By comparing with counts obtained from the negative control, the difference in the number of colonies before and after addition of the antimicrobial mat was calculated [10].

#### Viral test

AgNO_3_-PVP/APTMS/TEOS mat was soaked in water for 3 h, and then 10^−6^ Newcastle disease virus (LaSota strain) was incubated with the composite fibers for another 1 h. A set of five chicken embryos (11 days old) were prepared, and each was inoculated with 0.2 mL of the composite-incubated viral suspension, then placed at 37°C for 120 h. Similar experiments were set up with a suspension of pure Newcastle disease virus as the negative control.

Permanence of antimicrobial activity was investigated on the AgNO_3_-PVP/APTMS/TEOS fiber mat by using some pieces to repeatedly kill *E. coli* in 3-mL LB of 5 × 10^7^ cfu/mL. After each bacterial killing, the LB is carefully decanted and a fresh *E. coli* suspension added to the fiber samples. Aliquots from these cultures were plated on LB agar for cfu count to determine the number of surviving cells.

## Results and discussion

**Figure 1:**
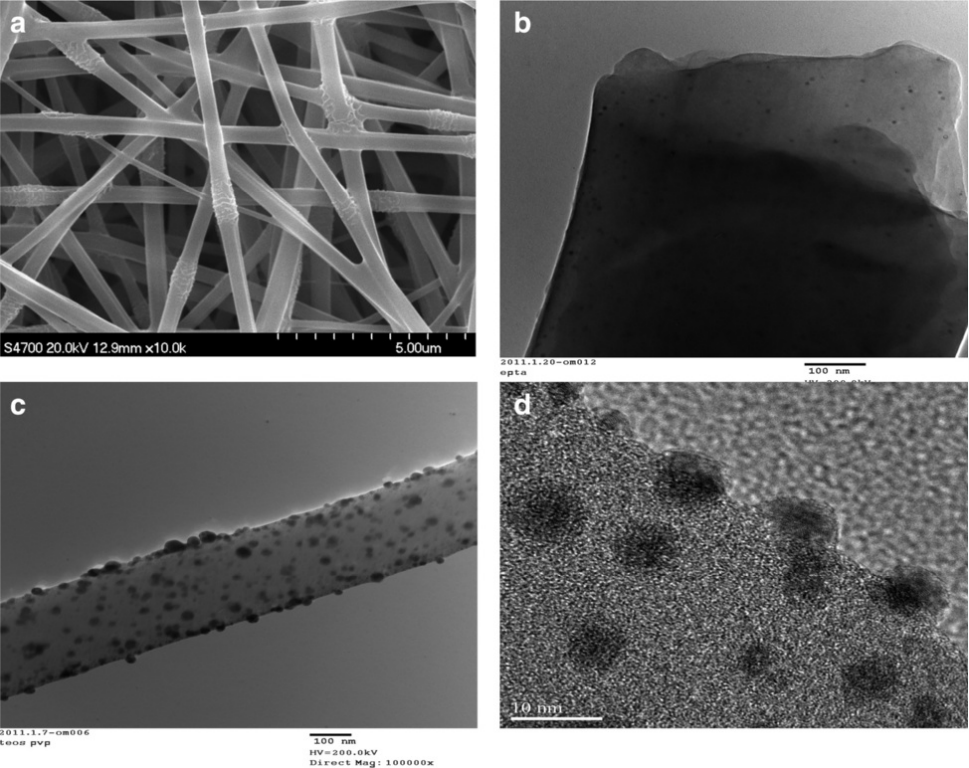
**Microscopic characterization of composite fibers.** (**a**) SEM image of AgNO_3_-PVP/APTMS/TEOS composite mat. (**b**) TEM image of AgNO_3_-PVP/APTMS/TEOS fiber (showing durable fiber; the black dots are small and highly dispersed silver metal particles). (**c**) TEM image of AgNO_3_-PVP/TEOS fiber (showing unstable fiber; the black dots are bigger and aggregated silver metal particles). (**d**) HR-TEM image of AgNO_3_-PVP/APTMS/TEOS fiber showing 5-nm crystal silver metal particles.

**Figure 2:**
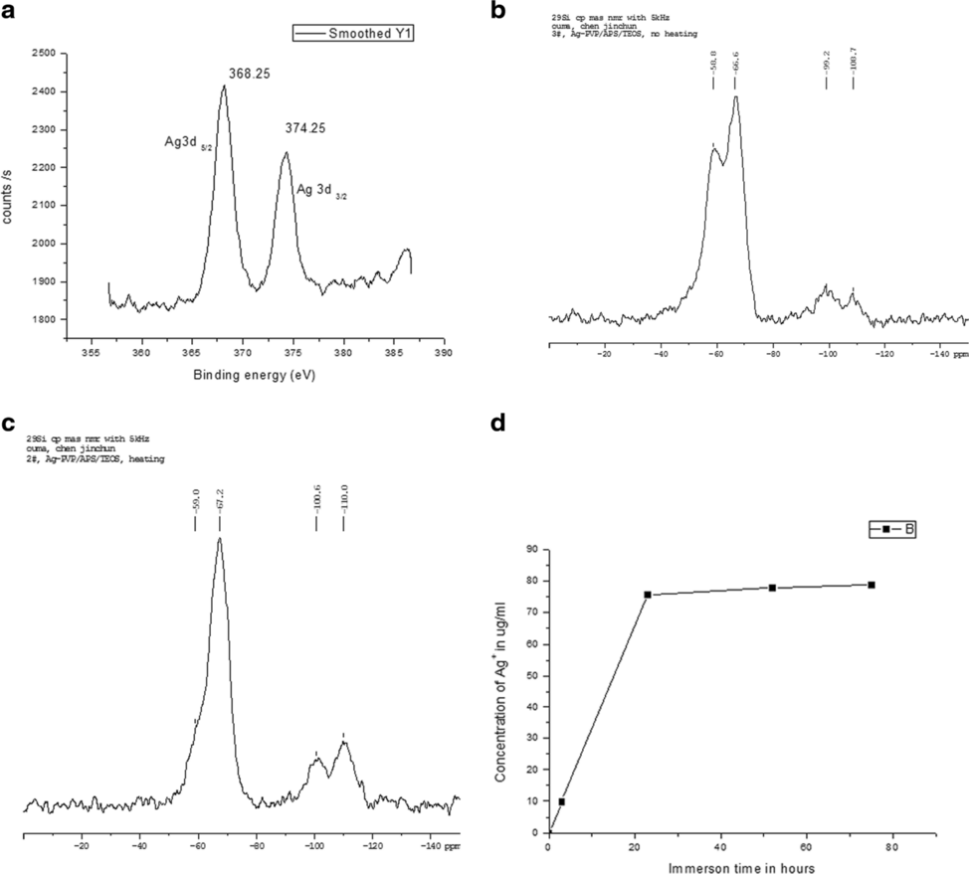
**Spectroscopic analyses of AgNO**_**3**_**-PVP/APTMS/TEOS composite.** (**a**) XPS spectrum of the composite’s silver species. (**b**) ^29^Si CP-MAS NMR of the composite before aging. (**c**) ^29^Si CP-MAS NMR of the composite after aging. (**d**) Composite’s silver release pattern.

Electrospinning is a process by which electrostatic force ejects a jet of fluid from droplets of a highly charged polymer solution or melt, then accelerates and deposits it on a collector target of opposite polarity to form a fiber. In this research, we electrospinned the solution of AgNO_3_-PVP/APTMS/TEOS (*v*/*v*) 3:2:1 to prepare a highly potent organoalkoxysilane-functionalized antimicrobial fiber mat. The composite is antimicrobial because of its silver and organoalkoxysilane contents. The as-spun fibers were left in air overnight for further hydrolysis and then aged at 60°C and 90°C for 5 h each and at 120°C for 2 h. Reduction of AgNO_3_ to metallic silver particles was achieved from the interaction of silver ions with various solution components especially PVP and heat treatment [11,12]. Two other composites were fabricated from PVP/APS/TEOS (*v*/*v*) 3:2:1 and AgNO_3_-PVP/TEOS (*v*/*v*) 3:2 as positive controls. The fabricated AgNO_3_-PVP/APTMS/TEOS was investigated with microscopic and spectroscopic analyses, and results are presented in Figures 1 and 2, respectively. Figure 1a shows the SEM image of the fiber mat, which reveals the composite’s porous and membranous morphology with fibers ranging from 300 to 500 nm in diameters. There was no significant shrinkage in the fibers’ diameter after heat treatments. TEM image of the composite in Figure 1b shows a fiber with uniformly distributed and highly dispersed Ag nanoparticles. Various functional groups of organoalkoxysilanes were reported to have a stabilizing effect on silver nanoparticles [13]. In this research, organoalkoxysilanes show influence on the size and dispersion of the composite’s silver metal particles. Highly dispersed smaller sized silver nanoparticles were synthesized when both APTMS and TEOS were added into the feed solution as depicted in Figure 1b, and bigger silver particles that tend to aggregate (Figure 1c) were observed when APTMS was not added. The silver particles of AgNO_3_-PVP/APTMS/TEOS are also much smaller than the 15- and 17-nm particles fabricated from the solution of silver nitrate, PVP, and TEOS by Jeon et al. [12] and Min et al. [14], respectively. We also observed that APTMS and TEOS bear importance on the morphology and structural stability of the fiber mat. Figure 1b shows the durable and transparent fiber with sharp smooth surfaces that were formed only in the presence of APTMS and TEOS, but when APTMS was not included in the feed solution, the unstable fiber (which cannot form fiber mat) shown in Figure 1c was fabricated. The HR-TEM image shown in Figure 1d reveals that well-crystallized, spherical silver particles (5 nm in size) were synthesized in the composite. Chemical identity of the composite’s silver was analyzed with XPS, and the results are shown in Figure 2a. Figure 2a shows binding energy peaks at 368.25 and 374.25 eV, which are typical of silver metal particles [15]. The results thus confirmed that the images seen in TEM and HR-TEM are actually silver metal nanoparticles. Figure 2b shows the ^29^Si cross*-*polarization magic angle spinning (CP-MAS) NMR analysis of the composite before heat treatment. It reveals peaks at −58.8, −66.6, −99.2, and −108.7 ppm assigned to T^2^, T^3^(SiO)_3_Si-R (a reaction product of APTMS with TEOS), Q^3^ silanol sites ((SiO)_3_SiOH), and Q^4^ silica sites ((SiO)_4_SiO), respectively [16]. Being T^3^(SiO)_3_Si-R the most abundant of all the reaction products suggests that majority of APTMS was consumed in polycondensation reaction, making its amino groups readily usable on the fiber mat. The varieties of silicon linkages of the reaction products, especially the T^3^(SiO)_3_Si-R, shown in Figure 2b form a network of skeleton in fibers. With rigidity and supports provided by the skeletons, fibers become stabilized and able to form membranous-porous fiber mat. The reason that fibers in Figure 1c are unstable is because they lack T^3^(SiO)_3_Si-R network support due to the absence of APTMS in their feed solution; hence, they could not form a fibrous mat. Figure 2c shows the ^29^Si CP-MAS NMR of the composite after aging. By depicting a decrease in T^2^ and an increase in all other reaction products especially T^3^, the results highlighted the importance of heat treatment in promoting further condensation of organoalkoxysilanes to reinforce and strengthen the fibers. Heat treatment also creates pores on fiber surfaces from the removal of water molecules produced during polycondensation. After aging, the composite becomes durable and less soluble in water.

The density of the composite’s surface amino groups as estimated by surface imine formation procedure [8] was 0.18 molecules/nm^2^. About 0.00079% of the total amino groups added to the feed solution are available as surface amino groups while 99.99921% is locked up within the composite’s body. The locked up amino groups become gradually exposed for antimicrobial action as the composite dissolves and worn out in the course of application. These results along with the observations made from NMR analyses confirm that amino groups are an integral part of the fibers. The pattern of silver ion release [9] from AgNO_3_-PVP/APTMS/TEOS fibers is illustrated in Figure 2d. The rapid buildup of high initial concentration of silver ions in the first 24 h followed by a steady and slow increase in concentration confers the fiber mat with quick and long-lasting antimicrobial activity.

**Figure 3:**
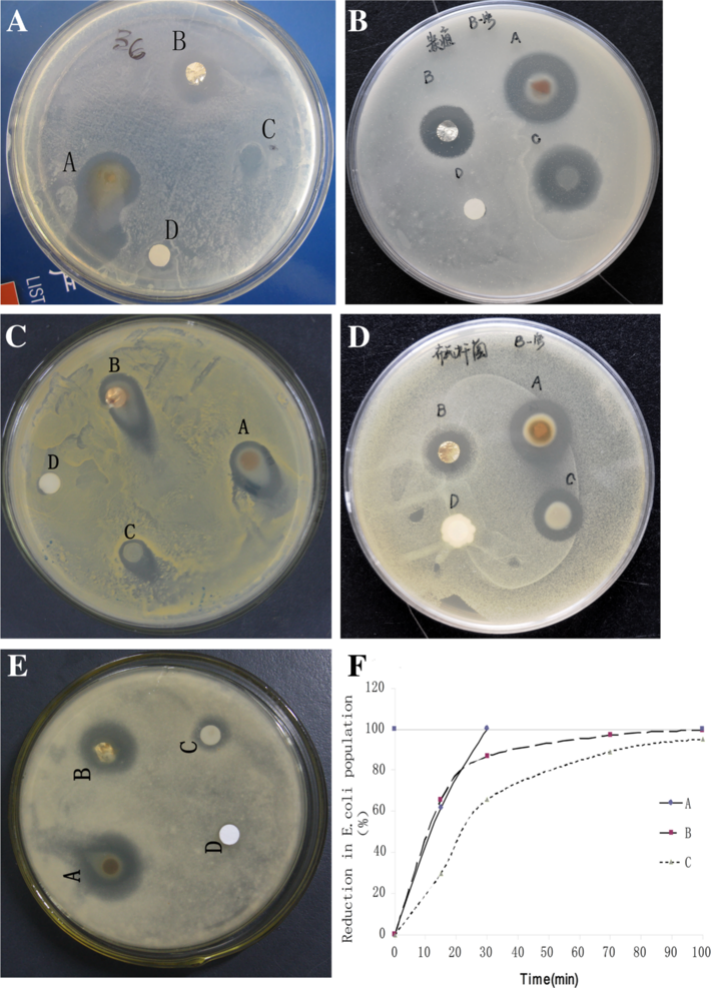
**Antimicrobial tests.** (**A** to **E**) Disk assays on *E. coli*, *B. anthracis*, *S. aureus*, *B. suis*, and *A. niger*, respectively. (**F**) Antimicrobial efficiency of composite mat on *E. coli*. A is AgNO_3_-PVP/APTMS/TEOS mat, B is AgNO_3_-PVP/TEOS, C is PVP/APTMS/TEOS, and D is a control clean filter paper.

Antimicrobial activity of the AgNO_3_-PVP/APTMS/TEOS mat was evaluated on gram-negative bacteria (*B. anthracis* CVCC40-221 and *E. coli* JM109), gram-positive bacteria (*S. aureus* ATCC6358P and *B. suis* strain 2 CVCC70-502), a fungus (*A. niger* ATCC10864), and Newcastle disease virus (LaSota strain) by disk diffusion, MIC, kinetic [10], and extended use assays. Results of antimicrobial tests are presented in Figure 3. Figure 3a,b,c,d,e shows results of the disk assay on *E. coli*, *B. anthracis*, *S. aureus*, *B. suis*, and *A. niger*, respectively. Diffusion of inhibitory substances from the composites prevented any microbial growth around AgNO_3_-PVP/APTMS/TEOS (sample A) and the two positive controls: AgNO_3_-PVP/TEOS (sample B) and PVP/APTMS/TEOS (sample C), creating clear zones of inhibition in all the plates. No zone of inhibition was formed around the control sample of clean filter paper (sample D). The area of inhibition zone around each sample was calculated by measuring its total size and then subtracting the sample size. The size of inhibition zones around sample A in *E. coli*, *B. anthracis*, *S. aureus*, *B. suis*, and *A. niger* are 27, 20, 14, 18, and 17 mm, respectively. With the same microbial sequence described for sample A, the inhibition zones around sample B are 9, 11, 17, 11, and 10 mm, respectively, while sample C has 3.7, 17, 8, 11, and 2.7 mm, respectively. These results suggest that all the fiber mat samples are antimicrobials. Judging from the sizes of inhibition zones, it is obvious that AgNO_3_-PVP/APTMS/TEOS fibers have superior antimicrobial potency compared to the two positive controls, thanks to the combined effect of its silver particles and organoalkoxysilanes. From the results, gram-negative bacteria appeared to be more sensitive to AgNO_3_-PVP/APTMS/TEOS than gram-positive bacteria, possibly due to the difference in their cell wall composition. Among the tested organisms, *E. coli* and *B. anthracis* showed the highest sensitivity toward AgNO_3_-PVP/APTMS/TEOS, while *B. suis* appeared to be more sensitive than *S. aureus* and *A. niger* shows sensitivity almost similar to that of *B. suis*. Sensitivity toward AgNO_3_-PVP/TEOS is almost similar among all the organisms under test. Inhibition zones formed around PVP/APTMS/TEOS fibers are due to the antimicrobial action of amino groups contained in the fine membrane fragments that easily dissolve and diffuse to the surrounding areas. Among all the organisms under test, *B. anthracis* is the most sensitive toward this composite. Sizes of inhibition areas remain unchanged after keeping the plates at room temperature for more than 6 months. This implies that the prepared composite has effective and long-term antimicrobial potency.

In the MIC test, the nanofiber mat sample weighing 0.3 mg provides the MIC for all the microorganisms under test. This MIC value is lower than those reported on other highly effective antimicrobial composite materials, such as the multiaction fibrous membrane containing apatite/Ag/AgBr/TiO_2_[4] and the dual-action AgBr/polymer composite [1] which achieved MIC at relatively high nanofiber concentrations of 0.6 mg and above. Not only our composite has a lower MIC value, but it is also silver efficient as the 0.3-mg fiber sample releases only 0.22 μg/mL of active silver to totally inhibit the microorganisms. This MIC silver concentration is much lower than the 37.65, 40, and 50 μg/mL [1,4,6] documented by other researchers. Attainment of this enhanced antimicrobial effectiveness with such small amount of nanofibers and active silver highlights the benefits of organoalkoxysilane functionalization and the advantages AgNO_3_-PVP/APTMS/TEOS has over previously reported antimicrobial composites. It is also important to note that both the qualitative and MIC test results attested to the composite’s broad-spectrum antimicrobial property on various groups of organisms.

From the results of kinetic studies presented on Figure 3f, AgNO_3_-PVP/APTMS/TEOS fibers show the fastest killing rate compared to positive controls. No bacterial growth was detected after 30 min of contact with AgNO_3_-PVP/APTMS/TEOS which means that the incubated cells have been inhibited and completely killed by the antimicrobial effect of the fibers. AgNO_3_-PVP/TEOS takes about 100 min to eradicate 99.67% of the incubated bacteria, while PVP/APTMS/TEOS reduces bacteria to 95.30%. As with the disc assay, results of kinetic tests also highlighted that all the composites are antimicrobials and AgNO_3_-PVP/APTMS/TEOS has stronger antimicrobial property than either of the two positive controls, further stressing the notion that synergy between silver and organoalkoxysilanes produces a composite with superior antimicrobial property. Moreover, in comparison to the previously reported composite materials which required 40 min, 50 min, and 24 h [1,12,17] to completely inhibit microorganisms, it is clear that AgNO_3_-PVP/APTMS/TEOS has better antimicrobial efficiency.

According to the results of antiviral analyses, only two out of the five fetuses inoculated with AgNO_3_-PVP/APTMS/TEOS-incubated viral suspension were infected, suggesting that the composite reduces viral infection capability by 60%. All the fetuses in the negative control were infected.

In repeated applications against *E. coli*, AgNO_3_-PVP/APTMS/TEOS shows cfu reductions of 98.0%, 99.6%, 99.0%, 99.0%, 99.3%, and 99.6% during the first, second, third, fourth, fifth, and sixth rounds of applications, respectively. The results suggest that the fiber mat has inherent antimicrobial property. Despite the repeated applications, the fibers’ ability to kill bacteria remains almost unchanged.

Functionalization with organoalkoxysilanes improves the antimicrobial effectiveness of AgNO_3_-PVP/APTMS/TEOS in a number of ways. First, organoalkoxysilanes aided in the synthesis of the very small-sized and highly dispersed silver nanoparticles in the mat; the two conditions are believed to enhance antimicrobial potency of composite silver particles [1,6]. Silver ions kill bacteria by creating holes and leakage in the cell [18], by deactivation of cellular proteins, and by causing shrinkage of cytoplasmic membrane and cellular response that permanently condenses DNA [19]. Second, APTMS equips the composite with surface amino groups to make the fibers adhesive and biocidal to microbial cells. Amino groups kill bacteria by capturing them and disrupting cellular functions [20] and by displacement of cations responsible for cell surface stability and integrity [21]. Furthermore, the synergistic antibacterial effect between the fibers' embedded silver and the surface amino groups and the surface amino groups greatly benefits the composite’s biocidal efficiency. By capturing bacteria, the surface amino groups could effectively decrease the distance between silver species and bacteria and facilitates the release of active silver into bacteria. Also, adhesiveness of amino groups may suppress bacteria by restricting their freedom of movement and confining them to one place. Third, the increased surface area formed by the membranous structure and porosity of the composite is also a critical factor for the high antimicrobial action of AgNO_3_-PVP/APTMS/TEOS. Availability of antimicrobial silver is not limited to the silver species in solution, but also those silver on fiber mat surfaces. The increased surface area therefore offers advantage of improving antimicrobial potency of active silver species over the conventional use of powder/aqueous silver ions. Both the porous structures and PVP content of fibers aid in water movement and leaching of silver from the composite mat. Moreover, the inherent ability of PVP to absorb and retain water makes the composite suitable for potential application in wound dressing where wound hydration and absorption of exudates are needed.

## Conclusions

The robustness of the electrospinning technique allows us to functionalize a silver-based composite with organoalkoxysilanes and fine tune the overall physical and chemical properties of the composite for improved antimicrobial potency and efficiency. Incorporation of organoalkoxysilanes improves the composite’s antimicrobial property as (1) hydrolytic polycondensation reaction of APTMS and TEOS creates silicon linkages to stabilize fibers and form a membranous mat of high porosity and surface area, (2) the silanes prevent aggregation of the synthesized silver particles, thereby facilitating the fabrication of very small-sized and highly dispersed silver metal particles in the composite, and in addition, (3) APTMS armed the composite fibers with amino groups to capture and kill microorganisms. Compared to other reported antimicrobial fibers, our composite shows superior antimicrobial performances. Test results also support the idea of dual-action mechanism of the fiber’s antimicrobial activity. The composite containing both silver and organoalkoxysilanes shows superior antimicrobial activity than those containing only silver or organoalkoxysilanes, thanks to the synergistic effect between various components of the composite. Further investigations showed that the fibers have inherent antimicrobial property and can be used repeatedly. The porous mat is biocidal to bacteria, fungus, and virus and may find applications in air filters, fabric linen for clothing, wound dressing, coatings, bioadhesives, and biofilms.

## Abbreviations

APTMS: 3-aminopropyl trimethoxysilane.

## Competing interests

The authors declare that they have no competing interests.

## Authors’ contributions

SU participated in the conception and design of the work and in the majority of the experiments, analyzed and interpreted the data of the experiment, and wrote the manuscript. YL participated in the conception and design of the experiment and in the antibacterial evaluation of the composite on *E. coli* and *S. aureus*. YW performed some of the fiber electrospinning experiments. GL participated in the conception and design of the experiment. JD carried out the antibacterial evaluation of the fibers on *B. anthracis* and *B. suis*. RX participated in the review of the manuscript. JC participated in the conception and design of the work and in the analysis and interpretation of the experimental data. All authors read and approved the final manuscript.

## Authors’ information

SU is a Ph.D. student at Beijing University of Chemical Technology, Beijing, China. YL is Master’s degree student at Beijing University of Chemical Technology, Beijing, China. YW is a Ph.D. student at Tsinghua University, Beijing, China. GL is a professor at Tsinghua University, Beijing, China. JD is a researcher at China Institute of Veterinary Drug Control, Beijing, China. RX is a Ph.D. student at Beijing University of Chemical Technology, Beijing China. JC is a professor at Beijing University of Chemical Technology, Beijing, China.
